# Care avoidance among homeless people and access to care: an interview study among spiritual caregivers, street pastors, homeless outreach workers and formerly homeless people

**DOI:** 10.1186/s12889-018-5989-1

**Published:** 2018-09-05

**Authors:** Hanna T. Klop, Kirsten Evenblij, Jaap R. G. Gootjes, Anke J. E. de Veer, Bregje D. Onwuteaka-Philipsen

**Affiliations:** 1 Department of Public and Occupational Health, Amsterdam Public Health research institute, Amsterdam UMC, VU University Amsterdam, P.O. Box 7057, 1007 MB Amsterdam, Netherlands; 2 Hospice Kuria, Valeriusplein 6, 1075 BG Amsterdam, Netherlands; 3 Netherlands Institute for Health Services Research (NIVEL), P.O. Box 1568, 3500 BN Utrecht, Netherlands

**Keywords:** Care avoidance, Interview study, Homeless persons, Trust, Understanding

## Abstract

**Background:**

Because of their poor health and social vulnerability, homeless people require specific care. However, due to care avoidance, homeless people are often not involved in care. This study aims to get insights into reasons for and kinds of care avoidance among homeless people and to provide suggestions to reach this target group.

**Methods:**

Semi-structured individual interviews were conducted among street pastors (*n* = 9), spiritual caregivers (*n* = 9), homeless outreach workers (*n* = 7) and formerly homeless people (*n* = 3). Participants were recruited by purposive sampling in the four major cities in the Netherlands (Amsterdam, Utrecht, Rotterdam, The Hague). The verbatim transcripts were analysed using thematic analysis.

**Results:**

The term *care avoidance* was perceived as stigmatizing. Care avoidance is found to be related to characteristics of the homeless person (e.g. having complex problems, other priorities) as well as of the system (e.g. complex system, conditions and requirements of organizations). The person-related characteristics suggestions to involve homeless persons include tailoring care and building relationships, which might even be prioritised over starting care interventions. Setting limits on behaviour without rejecting the person, and an attitude reflecting humanity, dignity and equality were also important factors in making care more accessible and lasting. As regards system-related characteristics, the suggestions include clear information and communication to homeless people who avoid care as being crucial in order to make care more accessible. Other suggestions include quiet and less busy shelters, a non-threatening attitude and treatment by professionals, self-reflection by professionals and finally a change of policy and legislation regarding available time.

**Conclusions:**

Reasons for care avoidance can be found in the interplay between both the individual and the system; measures to reduce care avoidance should be taken at both levels. These measures are centred on lowering the barriers to care inter alia by incorporating building trust and understanding into the care provided.

## Background

Because of their poor health and social vulnerability, people who are homeless require specific care. In this paper, we refer to homeless people as those who have unstable housing. They may use social day or night shelters including hostels, or avoid these accommodations and stay on the street. People living and sleeping on the streets or without fixed addresses also belong to this group. Unhealthy lifestyles, addiction and somatic morbidities such as lung diseases or cancer are common among the homeless [1–3] and morbidity rates are high [4–7]. Moreover, intellectual disabilities and mental health problems are more prevalent in this group when compared to the general population and many homeless people face financial problems [8–11]. Homeless people therefore need complex, multidimensional care. However, several studies have shown that homeless people have unmet care needs and poor access to care, and (partially) avoid or underuse this care [12–19]. Care avoidance is defined as partly or completely turning away from threat-related cues, which results in not being able or willing to be involved in care that is necessary [14, 20]. Care avoidance can be experienced as a problem by the homeless person (e.g. incomprehension or unmet needs) or the professionals (e.g. refusing necessary care or cancelling appointments). In this article, we use the term ‘care avoidance’, by which we mean ‘no access to care due to unwillingness or inability of homeless people or professionals, or the interaction between them’.

Care for the homeless can be divided in two types: i) social care for day and night shelters, housing, income and (social) activities on the one hand, and ii) medical care for addiction, mental and physical health issues on the other hand [21], which we call ‘healthcare’. Until now, most studies have focused on care avoidance in the context of healthcare. However, care avoidance can occur in broader areas, such as social issues, financial issues and housing issues. It is therefore crucial to cover all these areas by focusing on care avoidance in the context of both social and health care.

Although some evidence is available on unmet care needs of homeless people, poor access to care, and avoidance or underuse of care [12–19], studies into the nature of their care avoidance are scarce. This might be explained by the fact that it is hard to include homeless people who avoid care as a participant due to unfamiliarity with the care system [22, 23]. In the Netherlands, there is anecdotal evidence that spiritual caregivers and street pastors are better able to reach homeless people who avoid care than other professionals, due to activities such as establishing contact by visiting homeless people on the streets, and by providing help with practical matters. The nature of the contact between the homeless and both spiritual caregivers and street pastors is often characterized by low-threshold conversations and respectful relations without the consequence of being referred to care. Including them in a study could provide more insights into homeless people’s perspectives as well as those of care and healthcare professionals.

This study aims to get insight into the reasons for care avoidance, ways in which it occurs and how to deal with it by interviewing spiritual caregivers and street pastors. This provides more insight into the phenomenon of care avoidance according to people close to them, while at the same time more insight into methods is given for reaching this target group. The research questions were:

1. Why do spiritual caregivers and street pastors think homeless people avoid social care or healthcare?
2. Based on their experiences with reaching care avoiders, what suggestions do spiritual caregivers and street pastors have for making care accessible for homeless people who avoid care?

## Methods

### Design and participants

This study was designed as a qualitative study using in-depth interviews with spiritual caregivers and street pastors. A qualitative design was used to better understand the unexplored field of care avoidance, and for exploring the nature and context of care avoidance among the homeless. Differences between street pastors and spiritual caregivers concern religious background (street pastors have a religious background, spiritual caregivers not necessarily), aim (street pastors are specifically aimed at homeless people and spiritual caregivers can also be aimed at people in other contexts) and employment (street pastors are often employed by a church, spiritual caregivers often work within or for a healthcare organization). Participants were recruited using purposive sampling in the four major cities in the Netherlands (Amsterdam, Rotterdam, The Hague and Utrecht). We tried to ensure as much variation as possible in characteristics such as sex, age, religious beliefs, and city and recruited through the existing professional networks of the project team using the snowball method. Potential participants were contacted by telephone and informed by a letter about the topic and procedure. Beforehand, we intended to interview only spiritual caregivers and street pastors. During data collection, however, we found that besides spiritual caregivers and street pastors, some other homeless outreach workers such as street doctors or people who provide food also had very useful information on the topic. The information provided by these other homeless outreach workers corresponded with that of the street pastors and spiritual caretakers. This can be explained by the fact that they all use a similar working method: i.e. low-threshold contact. We therefore decided to include seven of them as well. In addition, we were given the opportunity to include former homeless people. In the first interviews with street pastors and spiritual caregivers, the researcher was told that interviewing former homeless perspective would enrich the perspective of street pastors and spiritual caregivers. We therefore decided to include three of them. In the end, 40 people were invited for interviews and 28 actually participated. Seven people did not respond, three were ineligible because they did not have contact with the target group, and two were not interested. In-depth interviews were held with nine spiritual caregivers, nine street pastors, seven other homeless outreach workers and three formerly homeless people. Characteristics of the participants are shown in Table 1. The number of street pastors that participated was high compared to the number of spiritual caregivers, especially considering the fact that the population of street pastors in the Netherlands is considerably smaller than the population of spiritual caregivers and the population of other homeless outreach workers. All participants were experienced in approaching people who avoid formal care, or were themselves formerly homeless persons who avoided (parts of) care. We followed the consolidated criteria guidelines for reporting qualitative studies (COREQ) [24]. This checklist consisted of three domains: (1) research team and reflexivity (among which personal characteristics and relationship with participants), (2) study design (among which theoretical framework, participant selection, setting, data collection), and (3) analysis and findings (among which data analysis and reporting). We took account of all items from the checklist when designing the study and article.

**Table 1:** Characteristics of participants (*n* = 28)

Interview	Profession	Sex	Age range (years)^a^	City^c^	Religious beliefs	Experience (years)^a, b^
#19	Spiritual caregiver	F	55–60	4	Catholic	1
#9	Spiritual caregiver	F	25–30	3	Humanist	3
#10	Spiritual caregiver	F	50–55	4	Protestant	7
#17	Spiritual caregiver	M	35–40	1	Humanist	8
#8	Spiritual caregiver	F	50–55	3	Protestant	9
#3	Spiritual caregiver	F	60–65	2	Protestant	10
#11	Spiritual caregiver	F	60–65	4	Protestant	10
#16	Spiritual caregiver	M	60–65	4	Protestant	15
#25	Spiritual caregiver	F	45–50	2	Protestant	15
#2	Street pastor	M	60–65	1	Protestant	3
#5	Street pastor	M	60–65	3	Catholic	5
#18	Street pastor	M	50–55	3	Protestant	6
#4	Street pastor	M	60–65	2	Protestant	9
#12	Street pastor	M	60–65	4	Protestant	9
#24	Street pastor	F	60–65	2	Protestant	11
#1	Street pastor	F	50–55	1	Protestant	13
#6	Street pastor	F	50–55	3	Protestant	14
#14	Street pastor	F	55–60	2	Catholic	14
#27	Formerly homeless person	M	50–55	1	None/NR^d^	1
#28	Formerly homeless person	M	45–50	2	None/NR	10
#22	Formerly homeless person	M	65–70	1	None/NR	10
#15	Homeless outreach worker	M	55–60	2	None/NR	1
#23	Homeless outreach worker	F	25–30	1	Protestant	8
#7	Homeless outreach worker	F	45–50	3	Catholic	11
#20	Homeless outreach worker	F	55–60	1	Protestant	12
#21	Homeless outreach worker	M	35–40	3	Protestant	13
#26	Homeless outreach worker	M	45–50	4	Protestant	14
#13	Homeless outreach worker	M	55–60	4	Protestant	26

^a^Mean age 52.8. Mean experience 9.8 years (formerly homeless people excluded)

^b^In case of formerly homeless people: years of living on the streets

^c^Each number represents one of the four major cities in the Netherlands

^d^*NR* Not relevant (formerly homeless people were not asked about their religious beliefs

### Ethics

Participants signed a consent form prior to the interview. This form contained information about the aim of the research and interview, global information about the interview topics, the confidentiality of the information provided and the guarantee of anonymity of quotes in publications. All participants received a gift voucher for their participation. Transcripts were anonymized after transcription to ensure anonymity of participants. Access to the data was limited to three researchers.

### Data collection

Data was collected from April 10 to October 6, 2017. All interviews were performed by the same researcher (HK), who was formerly employed by a homeless shelter and the Salvation Army, and is currently involved in palliative care for the homeless. Affinity with the topic of care avoidance arose when the researcher noted in previous studies that due to care avoidance a part of the homeless population is not involved in palliative care [25, 26]. All interviews were conducted at the participant’s location of choice, which was often the organization where the participant was employed, and in some cases at the participant’s home. The length of the interviews varied between 45 min and one hour. The interviews consisted of open questions and were guided by a semi-structured topic list, which was developed by two researchers (HK and BO) The topic list is shown in Table 2. This topic list evolved over time: some interview questions were deleted because they did not fit within the timeframe and the order of the subjects was slightly modified. Data saturation was discussed several times and after about 20 interviews no new information was found and no new themes emerged from the data.

**Table 2:** Topic list for semi-structured interviews

Care avoidance by homeless people • Current access to care and perceived problems • Extent of care avoidance • Signs of care avoidance • Causes of care avoidance • Characteristics of homeless people who avoid care • Care and aspects of care avoided	Care provided by participants • Care or guidance to homeless people • Reaching care avoiders and required expertise • Identified needs of homeless people who avoid care • Accessibility of care for target group • Examples of good and bad practices

### Data analysis

All interviews were audio recorded and transcribed verbatim. Written summaries were sent by e-mail to participants to evaluate correctness and validity. We followed the principles of thematic analysis [27]. First, transcripts were read and reread before analysis by HK and BO. Transcripts were then coded independently by two researchers (HK and KE) using Atlas.ti 7 and common themes were identified. Data from the first three interviews was analysed and discussed by HK and BO. After discussion, HK and BO decided that the themes already identified covered the important fragments from the transcripts, but that some codes had to be placed under other themes. Additionally, some topics needed to be highlighted more during the interviews that followed. Subsequently, all remaining transcripts were analysed and coded by HK. After that, codes were finally grouped into themes by HK and discussed with a second person (KE). Themes and interpretations were regularly and extensively discussed with BO, KE, JG, AV, HK through a draft report of the analysis and the themes identified.

## Results

After rereading the manuscripts and three stages of coding, common themes were grouped together following the research questions: factors that hamper accessible and appropriate care and possible solutions as suggested by respondents. For both categories a clear distinction could be made between themes related to characteristics of homeless people and characteristics of the care system. Furthermore, as several respondents commented on the term care avoidance, we considered this as a separate theme. We will address this theme first.

### Care avoidance – An ambiguous term

“Care avoidance” was considered to be stigmatizing by the participants, focussing primarily on the responsibility (or lack thereof) of homeless individuals rather than design issues of the care system related to care accessibility and usage. According to participants, several issues prevent homeless people from being involved in care including the way the care system functions and the fact that it can be difficult to access care. Participants therefore emphasized the importance of also looking at the inadequacy or inaccessibility of care.

*“So we’re building a system, and the homeless are left outside it because they either can’t fit or won’t fit in it – or both. And then we punish them by saying that they’re avoiding care.” (P15, homeless outreach worker)*

*“They aren’t care avoiders, they’re just careful about what care they accept. Saying it’s care avoidance is very negative. That says something about the person themselves, as if they don’t want any care. What I often see is that people look for care that suits them, which may not exist.” (P20, homeless outreach worker)*

It is therefore important to realize that care avoidance is about homeless people for whom care is not appropriate or inaccessible, and that it is not only a problem of homeless people. Participants gave many different reasons for care avoidance among the homeless, which we have divided into characteristics of homeless people and characteristics of the health system that hamper accessible or appropriate care. In reality, care avoidance is often a combination of these factors.

### Characteristics of homeless people that hamper accessible or appropriate care

The analysis showed six themes relating to homeless people themselves that might play a role in care avoidance. The five themes are shown in Table 3 below, each theme is illustrated with a quote. As a first theme, the majority of the participants mentioned that this group is diverse and that no stereotype exists (Table 3, Q1). There are people from all sorts of cultures, ages, with different medical and social problems, and with several reasons to avoid care. Despite this diversity some common denominators related to care avoidance did emerge from the interviews. As a second theme, a common factor among homeless people who avoid care is the complexity of their problems and the number of diagnoses and labels. (Table 3, Q2). Becoming homeless is often related to a variety of problems that are either medical or social in nature, such as a combination of psychiatric symptoms, addiction, intellectual disabilities, not having insurance, having debts, and housing deprivation. Also, being uninsured or undocumented was mentioned as a barrier to care. The stress and complexity of what is going on often make it difficult for homeless people to find solutions themselves. Besides, many homeless people have already been involved in healthcare for a long time, and have tried several social or medical services without success. They are often disappointed because they are not treated equally and they feel disparaged by professionals.

**Table 3:** Quotes on characteristics of homeless people that hamper accessible or appropriate care

Theme	No.	Quote
1. No stereotype	Q1	*“Care avoidance isn’t a single big group you can tackle in one go. The pattern varies hugely from one person to the next.” (P16, spiritual caregiver)*
2. Complexity of problems	Q2	*“I’d associate care avoidance with significant medical problems. They’ve also often been through a lot of psychotherapy and mental care already. They’ve seen it all before.” (P24, street pastor)*
3. Other priorities	Q3	*“If you’re homeless, you have to be ready to do things for your health that you don’t actually like. If they’d had normal lives, it maybe wouldn’t have been do difficult, but they don’t think it’s worth it. Scoring is often more important.” (P22, formerly homeless person)*
4. Maintaining self-control	Q4	*“I don’t think that they’re afraid to go to a doctor. They’re afraid that it’ll end up restricting their lives, you know? Suppose the doctor says you’ve got to be admitted to hospital... well, there’s no boozing or smoking in hospital. That lifestyle will be changed drastically – the doctor’s restricting what you can do, because you’ve got to go to hospital. They don’t want that. They don’t want to be tied down; they want to be able to respond when they think they need to. Maybe they’ll do nothing all day, but you never know – maybe the one time that they do have an appointment is just the moment when they could nick something or score or whatever. Yup, then they’re stuck with the appointment and they don’t think it’s right” (P22, formerly homeless person)*
5. Fear of stigmatization and treatment related to psychological or psychiatric problems	Q5	*“For mental things in particular, I think that people do avoid care. So it’s sometimes not so much care avoidance as denial and lack of an understanding of their illness as well. That’s often the case with psychiatric conditions. People who hallucinate but have a lot of difficulty admitting they’re hallucinations and who feel they’re being pigeon-holed. But if it’s a perception that you’ve got but they haven’t, then they feel they’re being accused of inventing things that they think are absolutely true. But, well, they don’t think there’s much point talking to a doctor anymore because they won’t be taken seriously.” (P6, street pastor)*

The third theme regarding care avoidance was that homeless people often have other priorities e.g. related to food or substance abuse due to their survival mode, which means living day to day and fulfilling basic needs (Table 3, Q3). Moreover, maintaining (self-)control in difficult circumstances (Table 3, Q4) was frequently mentioned as a fourth theme regarding reasons for avoiding care. As a fifth theme, it was stated that in cases of psychological or psychiatric problems homeless people might fear stigmatization and compulsory treatment and therefore stay away from care (Table 3, Q5).

### System-related characteristics that impede accessible or appropriate care

The analysis showed seven themes that concerned the relation between care and healthcare systems and care avoidance. The seven themes are shown in Table 4 below, each theme is illustrated with a quote. Overall, participants mentioned that many homeless people experience ‘the healthcare system’ as inaccessible and inappropriate, i.e. not meeting their needs and – directly or indirectly – leading to care avoidance. According to participants, the inaccessibility of the system is caused by several factors listed below.

**Table 4:** Quotes on system-related characteristics that hamper accessible or appropriate care

Theme	No.	Quote
System is complicated	Q7	*“It’s complicated finding the right organization as well. There are several options for your dole money or day care, for instance. Then there are the people from different situations, or from prison, where the probation service gets involved. Or there are people with addictions, where the addiction care service gets involved. Or the housing corporation – evictions or whatever – where a district team will sometimes have had a role too. And then you’ve got to find your way. So where do you start?” (P20, homeless outreach worker)*
Conditions and requirements of organizations	Q8	*“They’ve already said to start with that you should kick the drugs and then maybe we can help you with the mental problems. People can’t comply with those conditions – what’s going to replace them? They aren’t going to stop using because they’re suffering from sweaty feet. They’re going to keep using because they’re got an issue that they have to resolve,* i.e. *coping with daily life and the problem of what’s going on in their heads.” (P15, homeless outreach worker)*
System is inappropriate	Q9	*“Maybe it’s the level of education of the personal supervisors as well – that they’re simply not qualified enough to be able to deal with the entire spectrum of both mental and physical complaints. Ordinary staff, supervisors, they’re sitting there looking at a lump and wondering whether it means that a doctor is needed or not. They’re not really the people who should be dealing with those issues.” (P25, spiritual caregiver)*
Time pressure of professionals	Q10	*“The caregivers would also want to do more, but there simply isn’t the time. They only come along to tackle the issues of the moment and dole out medication. The homeless see perfectly well that they’re short of time too. They complain hugely about it, that the caregivers are often not really available.” (P25, spiritual caregiver)*
Attitudes of professionals	Q11	*“Many homeless people’s experience is that if they go anywhere – to a doctor or hospital or dentist – they get treated with a degree of suspicion.” (P4)*
Noisy and busy shelters	Q12	*“Some people actually find sleeping on the streets quite peaceful, because they think the care places are much too unsettled and too busy and too many other people snoring and far too much stuff that they don’t want.” (P8, spiritual caregiver)*
Patronizing and lacking participation	Q13	*“And one of these caregivers will then think, ‘I’m going to take you by the hand like a little kid and tell you how you have to do it.’ And if you do that to a fifty-year-old bloke who may have fought in a war or whatever, you’ll soon lose their respect. You mustn’t lay down the law for people. You can give them advice, though.” (P21, homeless outreach worker)*

The first theme refers to the complexity of the system for homeless people (Table 4, Q7). Care for homeless people is, as was stated, complex, multifaceted and scattered in its organization with a wide range of shelters, hostels, psychiatric institutions and nursing homes. In addition, consultation hours vary and helpdesks have limited and different opening hours. This is especially difficult for homeless people because of their psychosocial vulnerability including their intellectual and mental disabilities. Some specific problems related to the system were reported including regional bonding (which means that someone who is homeless is taken care of in the region they come from, this implies that care cannot be provided in other region), arranging things via the Internet, different locations for arranging different things, limited opening hours, waiting lists, protocols, rules and bureaucracy.

Secondly, participants reported that the conditions and requirements that people must adhere to in order to get access to care can be a reason for homeless people avoiding care (Table 4, Q8). These conditions are e.g. paying off debts in a certain way, detoxing, naming care needs and concrete questions, and going through processes quickly. Participants believed that enforcing such conditions and requirements meant care organizations were not sufficiently considering the needs or capabilities of homeless people.

Another theme that appeared from the interviews is that even when homeless people find access to the regular care system, the care they receive is often not appropriate, i.e. not adapted to their care needs. Their health problems often expand beyond the borders of individual somatic and psychiatric specialists, warranting a tailored and multidisciplinary approach in which interdisciplinary communication should be secured (Table 4, Q9).

Time pressure among professionals proved to be a recurrent barrier to providing appropriate care for the homeless. According to participants, current legislation and the way care is organized and financed contribute to heavy time pressure on professionals (Table 4, Q10). Professionals are bound to a specific amount of time, but as care for this population is more intensive and complex, it takes more time. As a result, there is little time for good conversations. Homeless people, however, generally consider real interest, attention and time for small talk and conversations to be very important. To achieve this, not only time but also the attitude of the professionals is important (Table 4, Q11). According to our participants, homeless people could experience professionals’ attitude as suspicious, asking questions unpleasantly, an attitude of knowing better, and applying their own standards. A lack of patience and creativity hampering finding solutions was also reported to be a barrier.

Many participants mentioned that homeless people perceive the day and night shelters as busy and overcrowded. Often, there is unrest (fights, disputes and security), little privacy and they have to be very careful when it comes to property. Moreover, professionals intend to immediately start arranging things and setting goals, which can increase stress. Homeless people, however, are in need of rest (Table 4, Q12). Avoiding busy shelters is a way of reducing stress.

Finally, while engagement and equality are very important values for homeless people, they are often left out in decision-making. According to participants, homeless people feel inferior, patronized and not taken seriously by professionals (Table 4, Q13). Consequently, they tend to avoid care professionals. According to the respondents, homeless people should have the right to be involved in formulating their needs and making decisions together with the professional despite their psychosocial limitations or issues.

#### Recommendations for facilitating access to homeless people who avoid care

Our second research question concerned suggestions of spiritual caregivers, street pastors, homeless outreach workers and formerly homeless participants for how professionals can improve the accessibility of care for homeless people. As indicated in the previous section, care avoidance has no single cause but is caused by factors related to the homeless people, the health system, and the interaction between them. Recommendations are aimed at a wide variety of professionals working in medical care, social assistance or a combination of these. In addition, each organization and professional has other sources, possibilities and limitations. Thus, for each organization and professional, another combination of recommendations will be relevant. In addition, a number of recommendations aimed at policy makers are addressed below. The suggestions for improving accessibility are categorized similarly. Firstly, we will discuss the suggestions related to the characteristics of homeless people themselves. Secondly, we will discuss the system-related suggestions. All themes are mentioned in Table 5, and illustrated by quotes.

**Table 5:** Quotes on suggestions for making care more accessible to homeless people who avoid care

Theme	Subtheme	No.	Quote
Characteristics of homeless people	Tailored care	Q1	*“Wouldn’t it be nice if the care system could offer more tailored solutions, saying ‘What would help you? And how can we work together so that here’s a place where you can get it. Get what would help you.’ Within reason, of course, but without saying it’s our way or the highway. Instead, you say there’s supervision but we’ll take a look at things with you to see what’s the best way of arranging it. So that it works for you.” (P1, street pastor)*
	Building relationships	Q2	*“Suppose I’ve found you and you very often sit at a particular time on a certain bench in the part, ‘cos that’s where you are with your two bags. Well, then you’ve not got to start by saying, ‘Come along with me.’ No, I think you’ve first got to ask if you can sit there with them. And maybe you don’t say anything else to start with. Getting someone to come and do what you want is still a long way down the road at that point.” (P19, spiritual caregiver)*
	Setting limits, but no rejection	Q3	*“We speak to people about their behaviour, we apply sanctions, but there’s one thing that we always make clear: you can always come back again. So it’s crucial that they know that the relationship will never be put under pressure. It can therefore mean that we ban people ten times, that they do something wrong ten times, but you can always come back again.” (P13, homeless outreach worker)*
	Humanity, dignity and equality	Q4	*“It’s pretty awkward for them; they’re never normal. So, well, they need in some way or other to be able to give and get something back. And so that they’ve got lots of skills and things that they can use, even if they are care avoiders.” (P23, homeless outreach worker)*
Characteristics of the system	Clear information, explanation and communication	Q5	*“Just be honest about it, like ‘It’s not possible right now with these waiting lists, but we’ll see what steps we can take that will really help you.’ And being straight up: ‘I can make this or that agreement with you now that we’ll take a look, because you want something to do. Right, I can call them now and see if there’s a place and I’ll call you, make an agreement with you, such and such a place and time, that’s when you’ll get the details from me.’ Short timelines, clear communication and clear agreements.” (P27, formerly homeless person)*
	Change of policy and legislation regarding to available time	Q6	*“My experience is that individual care providers are genuinely motivated, but that it’s often the structures of the institutions – imposed by legislation and regulations and driven by costs above all – that make it tricky. So it’s not the individual care providers, because they’re people who are really trying [to make the process less complex, more accessible, working from a relationship of trust, looking to see what the person themselves needs, communicating in understandable terms]. But, well, we need to set up the legislation and regulations and the funding so that it’s possible.” (P3, spiritual caregiver)*
	Quiet shelters	Q7	*“All the crisis shelters are in groups: several people sleeping in the same room. And it’s difficult there, it’s sometimes difficult to tailor the care there to suit... there’s just too little money for it, actually. A crisis shelter like that, there are just two of them for sixty people, so there’s no chance for any one-on-one supervision or whatever.” (P21, homeless outreach worker)*
	Attitude and treatment by professionals	Q8	*“We go to the station at five in the morning, at the times and moments that really suit them and not just during office hours. Care avoiders are often up and about at night, and they’ll sleep during the day, simply because sleeping in the daytime is safer. The other thing we do is start normal conversations with these people. So it’s not like ‘What do you need to get out of this situation’, but ‘How was your day? What are you going to do?’ And don’t think people won’t appreciate it, that it’s not part of the world they live in: it’s very much in their kind of world, because all day long they’ve got a caregiver or a cop or a guard hassling them, and never a normal person with a normal conversation.” (P15, homeless outreach worker)*
	Self-reflection of professionals	Q9	*“The caregivers also have to take a bit of a critical look at themselves. Like, well, I can’t do this; it isn’t working; there’s no click. We’re not making contact. So keep hacking away at those knots, and you mustn’t see it as failure.” (P27, formerly homeless person)*

## Related to the interaction between homeless persons and professionals

### Tailored care

To reach homeless people who avoid care, it is important to be able to offer personalized care. According to participants, tailored care means that professionals offer care that corresponds to the care needs of the homeless person. Additionally, tailored care implies that exceptions can be made when needed; professionals should be able to deviate from procedures or protocols, due to the complexity of needs and problems among people who are homeless (Table 5, Q1). A personal approach also involves attention to personal circumstances and capacity, e.g. coping skills or availability of time. Professionals have to understand that care cannot be limited to one discipline. In order to get a complete picture of the needs of homeless people avoiding care, it is important to explore the health, psyche, intellect, history, housing, finances, and other relevant social fields. According to participants, connecting all disciplines can provide tailored care. For this, it is important that professionals are well and specifically trained in the field of needs and preferences of homeless people, are able to find each other and ask each other for advice.

### Building a trusting relationship

The most frequently mentioned theme in the interviews was that in order to reach care avoiding homeless people, building a relationship between professionals and the homeless person is crucial (Table 5, Q2). An important requirement for building relationships is that it does not focus on providing care, but on the quality of the relationship. Conditions for building a trusting relationship include a consistent professional, and a feeling of having a connection between the homeless person and the professional. The professional should be easily approachable, reliable, keep in touch and have patience and time, as building trusting relationships may take a long time. Besides this, a ‘mediator’ can be helpful such as a street pastor, spiritual caregiver or case manager who can act in the care avoider’s interests during appointments.

### Setting limits, but no rejection

According to participants, it regularly occurs that care-avoiding homeless people fail to comply with agreements, misbehave or become angry or aggressive (Table 5, Q3). Participants stated that in such situations, it is very important that professionals do not reject the person. Professionals can accomplish this by seeing the behaviour as only one aspect of the person, by setting limits to the behaviour of their client, but at the same time offering the possibility to start again afterwards.

### Humanity, dignity and equality

Respect, humanity, dignity and equality are essential aspects in the attitude of a professional towards a homeless person, in order to ensure non-threatening and appropriate care (Table 5, Q4). For people who are homeless, it is especially important to feel respected. Humanity features a (more or less) equivalent exchange of personal information. Professionals can give examples of their personal experiences. Dignity also implies looking at what is important for homeless people. Equality is mainly about freedom of choice and not looking down at a homeless person as a professional.

## Related to characteristics of the system

### Clear information, explanation and communication

Information, explanation and communication are key to increasing the accessibility of care. The system is often experienced as complicated and homeless people can be overwhelmed by the difficult language used by health providers. It is crucial that homeless people are familiar with the rules and functioning of the healthcare system. They should be empowered to stand up for themselves (Table 5, Q5). This can be done concretely by providing information in simple and clear langue. Professionals must communicate clearly about the possibilities and impossibilities of their services and time schedule.

### Less busy day and night shelters

In order to improve accessibility of care, shelters should be more inviting. Day and night shelters are usually overcrowded and noisy and there is a lack of privacy: all reasons why homeless people tend to avoid care. To make day and night shelters more accessible, it is recommended for policy makers and managers in organizations that organizations offer smaller rooms and quieter geographical locations, even in urban environments (Table 5, Q7).

### Attitude and treatment: more patience, time and understanding

According to the participants, an open and friendly attitude of professionals towards homeless care avoiders is essential to improving accessibility (Table 5, Q8). Professionals should be aware that when dealing with care avoiders, more patience, time and understanding are needed than with the average person. In practice it is important that care providers create rest during appointments and treatment pathways, e.g. to start an appointment or treatment without too much pressure on commitment, engaging in small talk without providing care or to familiarize themselves with the background of their client. For doctors, this implies e.g. that several appointments are needed to fully map the problem and the history of the person. Given the limited time of doctors and high costs, it might be more feasible to deploy nurses to do these extensive intakes and let them mediate the doctor’s appointment, or to engage another mediator who joins homeless people to the appointment, for example a street pastor, spiritual caregiver or other homeless outreach worker.

### Self-reflection by professionals

Many participants stated that professionals need sufficient self-awareness and must be critical of their own actions (Table 5, Q9). The way a professional sees things can be very different to the care avoider. A professional must be aware of this and realize that their standards and expectations may not always be the same as those of the homeless person. An open attitude is crucial. Self-reflection also means acknowledging mistakes and being honest about their professional limitations.

### Change of policy and legislation with regard to available time of professionals

Finally, participants indicated that the time available for care is too limited (Table 5, Q6). This is mainly due to current legislation and regulations and the policies of organizations. In order to provide effective and appropriate care, more intensive care is needed, which takes more time.

## Discussion

Spiritual caregivers, street pastors, homeless outreach workers and formerly homeless people, experienced the term ‘care avoidance’ to be stigmatizing. Care avoidance seemed to focus only on the homeless and not on the system, while inaccessibility of the system is a barrier to involvement in care. This study shows that care avoidance was not only related to the characteristics of homeless people (e.g. complex problems, other priorities), but also to system-related characteristics (e.g. complicated system, conditions and requirements of organizations), both impeding care involvement. This supports the findings of Schout et al. [28], who introduced the term ‘care paralysis’, meaning the inability of professionals in social services to help people with multiple and complex problems. By using both terms simultaneously, i.e. care avoidance and care paralysis, it is more clear that not only characteristics of the homeless but also from the system must be addressed in order to improve care avoidance. Moreover, this might be experienced as less stigmatizing.

Previous studies in the field of care avoidance, although carried out among other populations, show similar reasons for care avoidance, such as mistrust or a lack of confidence, negative evaluations of the quality of care and previous negative experiences with care providers’ communication styles and seeking healthcare [29–33]. Additionally, several studies also reported a low perceived need to seek medical care as a reason to avoid care [30, 32]. However, existing literature also provides some reasons for avoiding care that were indeed confirmed by our study but were less evident such as a fear of serious illness or of thinking about dying [30, 33, 34], insured lack of (health) insurance and being illegal [31, 32].This was most likely due to the focus of the interviews which was more on the complexity of various problems among the homeless, as a result of which a lack of health insurance and illegality appear less prominently as reasons for care avoidance. Since the reasons for care avoidance identified by this study are similar to the reasons previously reported for other populations, the question can be raised whether care avoidance among the homeless is essentially different from care avoidance among other populations. Nevertheless, the multifaceted nature of the problems, the focus on psychosocial barriers, and the need for a multidisciplinary approach, respect, understanding and trust do distinguish this group from other populations. Ye, Shim & Rust provide evidence for the focus on psychosocial barriers to care among this target group. They mentioned that people with serious psychological distress were more likely to report psychosocial barriers to care [34].

Participants made suggestions that could help reach care avoiders, related to characteristics of both the homeless and the system. The person-related characteristics include tailoring care and building relationships at an early stage. It is often necessary to build a trustful relationship before care can be provided. Meeting the practical needs of the homeless person is key in building this relationship. When care is provided, the needs of homeless must be leading [35, 36]. Furthermore, setting limits to behaviour without rejecting the person, and an attitude reflecting humanity, dignity and equality were also important factors in making care more accessible and long-lasting. Regarding system-related characteristics, clear information and communication to homeless people who avoid care are important for making care more accessible. Other system-related suggestions include quiet and less busy shelters, unthreatening attitudes and treatment by professionals, self-reflection by professionals and finally a change of policy and legislation regarding available time. Other studies in care for the homeless have also reported the need for respect, understanding, trust and easier access to health services [37–39]. Not being able or willing to be involved in care has several reasons, which are mainly “demands, thresholds and fragmentation of services, which hinder the accessibility of healthcare”, and more specifically “disputes, conflicts, suspicions about the intentions of healthcare professionals and a mismatch between expectations and provision of care” [14]. Our findings confirm a previous study on care avoidance in psychological care by amongst others homeless people, which highlighted the importance of establishing contact and winning trust [14] and reported that care avoidance is often caused by the interplay between characteristics of both the clients and the system. It is important to notice that many of the solutions can only be successful if health care professionals realize that they have agency and have an important role in achieving improvements. Thinking beyond the individual causes of care avoidance, i.e. either related to the homeless person or the system, but also having attention for the interplay between those causes is crucial.

While several respondents of our study reported the term care avoidance to be problematic, respondents of another study did not report this [14]. This does raise the question whether we should discard the term ‘care avoidance’ and use more neutral terminology related to the accessibility of care for different target groups, or at least emphasize that care avoidance can be rooted in both personal and system characteristics.

To our knowledge, this study is the first to explore the reasons for avoiding care and the way to overcome this from the perspective of spiritual caregivers, street pastors and homeless outreach workers, and is supported by experiences of formerly homeless people. It is a strength of our study that we were able to expand the respondent group, i.e. including also homeless outreach workers and formerly homeless people. Together, the participants interviewed were people who view both the perspective of the target group and the world of professional care. It therefore gives practical suggestions for improving accessibility of care based on the non-threatening working methods used by these participants. This was confirmed in the interviews with the formerly homeless participants, who stated that the spiritual caregivers and street pastors were the people who were most closely attached and committed to them, and who understood them well. A limitation of our study was the different nature of care that street pastors and spiritual caregivers provided in comparison with other care providers in medical and social care, who often experience more obligations and more time pressure. This might make it difficult for the latter to implement the suggestions for improvement. However, several of the recommendations can be implemented in any event, e.g. attitude. Moreover, although building relationships might require an investment at first, this may strengthen relationships and contributes to providing more appropriate and more accessible care.

## Conclusions

Reasons for care avoidance turned out to be a combination of characteristics of homeless individuals as well as characteristics of the system and the competences of the professionals working with homeless people. Measures to reduce care avoidance should be taken at both levels. While changes in the organization of healthcare need measures from policy makers, medical and social care professionals can also help reduce care avoidance. A low-barrier method such as that of street pastors, spiritual caregivers and homeless outreach workers, involves building relationships by building trust, showing understanding and being with the homeless person. By paying attention, the professional can discover what these values mean for the homeless and what this implies for providing care and starting a care pathway.

## Abbreviations

AV: Anke de Veer
BO: Bregje Onwuteaka-Philipsen
COREQ: Consolidated criteria guidelines for reporting qualitative studies
HK: Hanna Klop
JG: Jaap Gootjes
KE: Kirsten Evenblij
NR: Not relevant
P[number]: Participant [number]
Q[number]: Quote [number]

## References

[1] Fazel S, Geddes JR, Kushel M. The health of homeless people in high-income countries: descriptive epidemiology, health consequences, and clinical and policy recommendations. Lancet (London England). 2014; 10.1016/S0140-6736(14)61132-6. 10.1016/S0140-6736(14)61132-6 PMC4520328 25390578

[2] Chau S, Chin M, Chang J, Luecha A, Cheng E, Schlesinger J (2002). Cancer risk behaviors and screening rates among homeless adults in Los Angeles County. Cancer Epidemiol Biomarkers Prev.

[3] Hwang SW (2001). Homelessness and health. CMAJ.

[4] Scott J, Gavin J, Egan AM, Avalos G, Dennedy MC, Bell M (2013). The prevalence of diabetes, pre-diabetes and the metabolic syndrome in an Irish regional homeless population. QJM.

[5] Oliveira Lde P, Pereira ML, Azevedo A, Lunet N (2012). Risk factors for cardiovascular disease among the homeless and in the general population of the city of Porto, Portugal. Cad Saude Publica.

[6] Schanzer B, Dominguez B, Schrout PE, Caton CLM (2007). Homelessness, health status, and health care use. Am J Public Health.

[7] Slockers MT, Nusselder WJ, Rietjens J, van Beeck EF. Unnatural death: a major but largely preventable cause-of-death among homeless people? Eur J public health. 2018; 10.1093/eurpub/cky002. 10.1093/eurpub/cky002 29415211

[8] Van Straaten B, Schrijvers CTM, Van der Laan J, Boersma SN, Rodenburg G, Wolf JRLM, et al. Intellectual disability among Dutch homeless people: prevalence and related psychosocial problems. PLoS One. 2014; 10.1371/journal.pone.0086112. 10.1371/journal.pone.0086112 PMC3897643 24465905

[9] Fazel S, Khosla V, Doll H, Geddes J. The prevalence of mental disorders among the homeless in western countries: systematic review and meta-regression analysis. PLoS Med. 2008; 10.1371/journal.pmed.0050225. 10.1371/journal.pmed.0050225 PMC2592351 19053169

[10] Lebrun-Harris LA, Baggett TP, Jenkins DM, Sripipatana A, Sharma R, Hayashi AS (2013). Health status and health care experiences among homeless patients in federally supported health centers: findings from the 2009 patient survey. Health Ser Res.

[11] Ball SA, Cobb-Richardson P, Connolly AJ, Bujosa CT, O'neall TW (2005). Substance abuse and personality disorders in homeless drop-in center clients: symptom severity and psychotherapy retention in a randomized clinical trial. Compr Psychiatry.

[12] Hwang SW, O'Connell JJ, Lebow JM, Bierer MF, Orav EJ, Brennan TA (2001). Health care utilization among homeless adults prior to death. J Health Care Poor Underserved.

[13] Canavan R, Barry MM, Matanov A, Barros H, Gabor E, Greacen T (2012). Service provision and barriers to care for homeless people with mental health problems across 14 European capital cities. BMC Health Serv Res.

[14] Schout G, de Jong G, Zeelen J. Establishing contact and gaining trust: an exploratory study of care avoidance. J Adv Nurs. 2010; 10.1111/j.1365-2648.2009.05171.x. 10.1111/j.1365-2648.2009.05171.x 20423415

[15] Lamb V, Joels C (2014). Improving access to health care for homeless people. Nurs Stand.

[16] Baggett TP, O'Connell JJ, Singer DE, Rigotti NA (2010). The unmet health care needs of homeless adults: a national study. Am J Public Health.

[17] Palepu A, Gadermann A, Hubley AM, Farrell S, Gogosis E, Aubry T, et al. Substance use and access to health care and addiction treatment among homeless and vulnerably housed persons in three Canadian cities. PLoS One. 2013; 10.1371/journal.pone.0075133. 10.1371/journal.pone.0075133 PMC3790780 24124470

[18] Krausz RM, Clarkson AF, Strehlau V, Torchalla I, Li K, Shuetz CG (2013). Mental disorder, service use, and barriers to care among 500 homeless people in 3 different urban settings. Soc Psychiatry Psychiatr Epidemiol.

[19] Argintaru N, Chambers C, Gogosis E, Farrell S, Palepu A, Klodawsky F, Hwang SW. A cross-sectional observational study of unmet health needs among homeless and vulnerably housed adults in three Canadian cities. BMC Public Health. 2013; 10.1186/1471-2458-13-577. 10.1186/1471-2458-13-577 PMC3691921 23764199

[20] Byrne SK (2008). Healthcare avoidance: a critical review. Holist Nurs Pract.

[21] Van Laere I, Withers J (2008). Integrated care for homeless people--sharing knowledge and experience in practice, education and research: results of the networking efforts to find homeless health workers. Eur J Pub Health.

[22] Van Straaten B, Rodenburg G, Van der Laan J, Boersma SN, Wolf JRLM, Van de Mheen D (2016). Substance use among Dutch homeless people, a follow-up study: prevalence, pattern and housing status. Eur J Pub Health.

[23] Van Straaten B, Rodenburg G, Van der Laan J, Boersma SN, Wolf JRLM, Van de Mheen D. Changes in social exclusion indicators and psychological distress among homeless people over a 2.5-year period. Soc Indic Res. 2016; 10.1007/s11205-016-1486-z. 10.1007/s11205-016-1486-z PMC5785592 29398768

[24] Tong A, Sainsbury P, Craig J (2007). Consolidated criteria for reporting qualitative research (COREQ): a 32-item checklist for interviews and focus groups. Int J Qual Health Care.

[25] Klop HT, de Veer AJE, van Dongen SI, Francke AL, Rietjens JAC, Onwuteaka-Philipsen BD. Palliative care for homeless people: a systematic review of the concerns, care needs and preferences, and the barriers and facilitators for providing palliative care. BMC Palliat Care. 2018; 10.1186/s12904-018-0320-6. 10.1186/s12904-018-0320-6 PMC5914070 29690870

[26] Klop HT, van Dongen SI, Francke AL, de Veer AJE, Rietjens JAC, Gootjes JRG, Onwuteaka-Philipsen BD. The views of homeless people and health care professionals on palliative care and the desirability of setting up a consultation service: a focus group study. J Pain Symptom Manag. 2018; 10.1016/j.jpainsymman.2018.05.026. 10.1016/j.jpainsymman.2018.05.026 29885872

[27] Braun V, Clarke V (2006). Using thematic analysis in psychology. Qual Res Psych.

[28] Schout G, de Jong G, Zeelen J (2011). Beyond care avoidance and care paralysis: theorizing public mental health care. Sociology.

[29] Leyva B, Taber JM, Trivedi AN. Medical care avoidance among older adults. J Appl Gerontol. 2017; 10.1177/0733464817747415. 10.1177/0733464817747415 29249189

[30] Kannan VD, Veazie PJ (2014). Predictors of avoiding medical care and reasons for avoidance behavior. Med Car.

[31] Spleen AM, Lengerich EJ, Camacho FT, Vanderpool RC. Health care avoidance among rural populations: results from a nationally representative survey. J Rural Health. 2014; 10.1111/jrh.12032. 10.1111/jrh.12032 PMC3882339 24383487

[32] Taber JM, Leyva B, Persoskie A (2015). Why do people avoid medical care? A qualitative study using national data. J Gen Intern Med.

[33] Yousaf O, Grunfeld EA, Hunter MS (2015). A systematic review of the factors associated with delays in medical and psychological help-seeking among men. Health Psychol Rev.

[34] Ye J, Shim R, Rust G (2012). Health care avoidance among people with serious psychological distress: analyses of 2007 health information National Trends Survey. J Health Care Poor Underserved.

[35] Tsemberis SJ, Moran L, Shinn M, Asmussen SM, Shern DL (2003). Consumer preference programs for individuals who are homeless and have psychiatric disabilities: a drop-in center and a supported housing program. Am J Community Psychol.

[36] Tsemberis S, Asmussen S (1999). From streets to homes: Pathways to Housing consumer preference supported housing model. Alcohol Treatment Q.

[37] Roche MA, Duffield C, Smith J, Kelly D, Cook R, Bichel-Findlay J, et al. Nurse-led primary health care for homeless men: a multimethods descriptive study. Int Nurs Rev. 2017; 10.1111/inr.12419. 10.1111/inr.12419 29266302

[38] Van den Berk-Clark C, McGuire J (2014). Trust in Health Care Providers: factors predicting trust among homeless veterans over time. J Health Care Poor Underserved.

[39] Irestig R, Burström K, Wessel M, Lynöe N. How are homeless people treated in the healthcare system and other societal institutions? Study of their experiences and trust. Scand J Public Health. 2010; 10.1177/1403494809357102. 10.1177/1403494809357102 20056785

